# Understanding the flower rate of litchi (*Litchi chinensis* Sonn.) through machine learning predictive models

**DOI:** 10.3389/fpls.2026.1865699

**Published:** 2026-10-27

**Authors:** Zuanxian Su, Lunyang Liu, Shanguo Ou, Xiaodan Peng, Guixiang Zhang, Yunqi Li, Houbin Chen

**Affiliations:** 1 Guangdong Litchi Engineering Research Center, College of Horticulture, South China Agricultural University, Guangzhou, China; 2 Guangdong Provincial Doctoral Workstation, Qiyuan Agricultural Development Co., Ltd., Gaozhou, China; 3 Changchun Institute of Applied Chemistry, Chinese Academy of Sciences, Changchun, China; 4 Guangzhou Ecological and Agricultural Meteorological Center, Guangzhou, China; 5 Greater Bay Area (GBA) Meteorological Intelligent Equipment Research Center/Greater Bay Area (GBA) Academy of Meteorological Research, Guangzhou, China; 6 Department of Polymer Materials and Engineering, College of Materials and Metallurgy, Guizhou University, Guiyang, China

**Keywords:** chilling accumulation, flower rate, heat accumulation, machine learning, phenological phase, SHAP

## Abstract

Flower rate is an important indicator dominating the harvest litchi fruit of high quality, which has complex interactions among inherent characteristics of individual trees, climate, soil, fertilizer and responsive managements. Quantitative prediction of the flower rate utilizing machine learning algorithms is feasible, so to determine important features and to implement appropriate management responses. In this study, based on a comprehensive collection of data in climate conditions, cultivar types, tree ages, phenological phases and historical flowering patterns coupled with the flower rate of litchi spanning the years from 2009 to 2020 in the southern region of China, a set of robust predictive models were constructed. Binary classification models delivered an AUC in the range from 0.73 to 0.90, and regression models achieved the coefficient of determination (R^2^) from 0.64 to 0.73 in the blind tests on annual data. SHAP analysis revealed that the date of floral white millet emergence, the accumulation of chilling hours below 9, 10 and 13 °C in the process of flower induction and their annual average, ages for trees, and the mean atmospheric relative humidity per hour in the process of flower induction are the most important features. Through the exploration of their positive or negative contributions to the floral induction, stimulate the timely emergence of “white millet” through irrigation, pruning, and foliar fertilization, practical guidance can be obtained and validated through expertized rationality. This study provided solid guidance to improve the flower rate of litchi that can promote the efficient responsive management to harvest litchi fruit of high quality.

## 1 Introduction

The formation of flowers, a critical determinant of fruit tree yield, has spurred research interest in the impacts of global climate change on flowering rates in recent years. Two primary approaches predict preharvest yield: crop growth models, which require extensive inputs and calibration (Jha et al., 2021), and machine learning models, which infer relationships from data without presupposed hypotheses (Behmann et al., 2015). Despite not necessitating deep mechanistic understanding, machine learning approaches are increasingly applied in agriculture to model complex interactions and manage variables crucial for yield estimation (Cai et al., 2019; Liakos et al., 2019), environmental sustainability (Saha et al., 2021) and the impacts of climate change (Vogel et al., 2019). Litchi is a valuable tropical fruit tree grown in over 20 countries, contributing significantly to local economies. It’s yield and quality can be significantly affected by climate and environment changes, and their solid exact impacts may be unveiled through the construction of machine learning based predictive models.

The nutritional benefits, distinctive tastes, and vibrant colors make litchi have expanding international market (Hu et al., 2022). On the supply chain of planting, improving cultivation practices and maximizing flower rates are crucial (Rising and Devineni, 2020; Rizzo et al., 2022). Fundamentally, deep understanding the flower rate from influential factors should help better management decisions (Mathan et al., 2016). Floral formation, a complex physiological process integrating genetic, morphological, and environmental cues, is pivotal for fruit tree yield. While morphological maturity (e.g., autumn shoots) and physiological states (high C/N ratio, starch accumulation) are prerequisites for floral induction and differentiation (Menzel, 1983; O’Hare, 2002; Yuan et al., 2009; Zhang et al., 2014), these processes are dynamically modulated by environmental factors. Temperature and water availability, for instance, regulate flowering through hormone signaling and carbon metabolism, with strategies like moderate autumn water stress enhancing C/N ratios and flower intensity (Stern et al., 1998; Carr and Menzel, 2014). However, direct physiological monitoring is impractical for large-scale orchards. Thus, deciphering non-linear environmental-physiological interactions requires machine learning to translate accessible climate data into actionable insights for precision management, particularly for economically vital crops like litchi.

Temperature fluctuations critically influence litchi growth and reproduction, with climate change exacerbating extreme weather events that induce irregular flowering and yield losses (Pék and Helyes, 2004; Hatfield et al., 2011). While conventional management strategies, such as water regulation, can mitigate some temperature-induced stresses (Hatfield and Prueger, 2015), precise timing is key. Our prior study revealed that tree traits and specific environmental windows determine floral induction timing (Su et al., 2023a). However, traditional statistics struggle to disentangle complex, high-dimensional interactions among tree status, soils, and climate variables. Thus, a data-driven approach is imperative to identify critical thresholds governing flower rates, enabling targeted orchard management.

To explore these complex interactions in plating, various machine learning algorithms can be selected, but the optimal one is strongly depending on factors like dataset size, data noise, model accuracy, complexity and computational cost (Enke and Mehdiyev, 2012). Random Forest (RF) has been proven an effective algorithm to predict crop yields (Shahhosseini et al., 2019, 2021), they normally trained model using data from previous seasons then predict yields in the next year so to minimize the impacts from dramatic climate changes (Desloires et al., 2023). Meanwhile, the development of Shapley additive explanation (SHAP) analysis (Lundberg and Lee, 2017) together with RF algorithm (Breiman, 2001) make the interpretation of models become powerful. The SHAP value for each feature allow the visualization of correlations at case level (Parsa et al., 2020), successfully explored cotton yield and its determinants (Kaur Dhaliwal et al., 2022) and crop yield and key factors for response management (Jones et al., 2022). It is interesting to know whether the response management for the flower rate of litchi can be improved through the integration of these ML based predictive models.

Therefore, this work is to assess the efficiency of machine learning predictive models for the flower rate of litchi, then to determine important features for response management. The dataset includes information on phenology, temperature fluctuations, humidity levels, and chilling/heat accumulation below or above a specified threshold during the induced flowering of litchi. Implicit and explicit prediction models for classifying the flowering state (Flr.State) were developed using (RF) and Linear Discriminant Analysis (LDA). In addition, this work evaluated the importance of the selected features using RF and SHAP to provide guidelines for response management to harvest litchi with high flower rate.

## 2 Datasets and methods

### 2.1 Datasets

Raw data for the perennial litchi cultivars “Guiwei (GW)”, “Nuomici (NMC)”, “Huaizhi (HZ)”, and “Feizixiao (FZX)” were obtained from the SCAU Wushan Campus Teaching & Research Base (23.164°N, 113.366°E) in Guangzhou City. The plants may have one to three flushes post-harvest, with the final flush matured in mid-October. The number of trees for these four cultivars were 33, 12, 20, and 11, respectively. Phenological data, such as the date of final autumn flush maturation (DOFM), date of floral white millet emergence (DOFE), and flowering proportion, were recorded for all plants. Plants were classified into the DOFM stage when 75% of the previous autumn flushes reach maturity, and entries for the DOFE stage were determined by the emergence of 25% of floral white millets. Given the rapid emergence of floral white millets within a few days, the 25% threshold is utilized to enhance the accuracy of discrimination for the DOFE stage. A schematic diagram illustrating the litchi flowering and fruiting process was presented in **Figure 1**.

**Figure 1:**
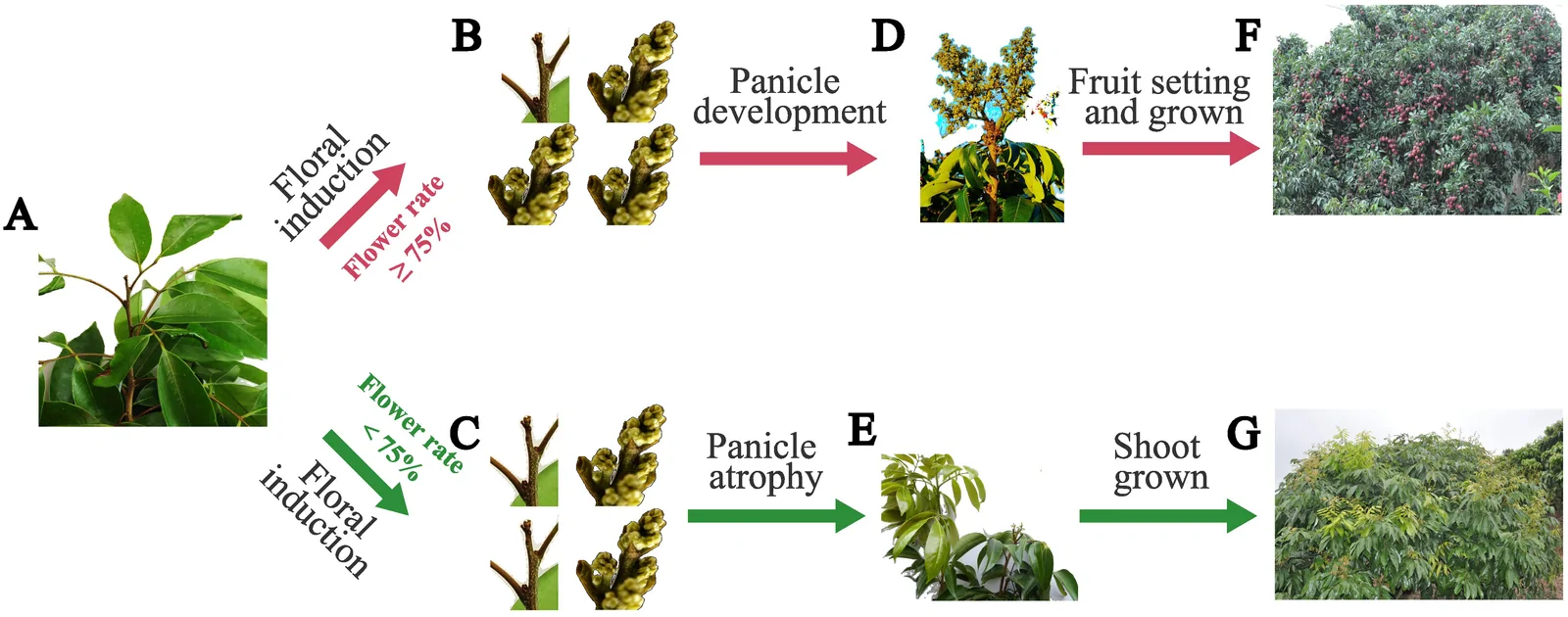
Schematic diagram of litchi flowering and fruiting process. **(A)** the last batch of mature shoots in autumn. **(B)** floral shoots with flower rate ≥ 75%. **(C)** floral shoots with flower rate < 75%. **(D)** generative panicle. **(E)** leafy panicle. **(F)** high yielding plant. **(G)** low yielding plant.

Hourly air temperature and humidity data were also collected from October to March each year from 2009 to 2020 using a Two-Channel Data Logger (SSN23 version, YuWen Corporation, Shenzhen) positioned 1.5 meters above the ground in a Stevenson screen. The daily mean temperature and humidity for the period from 2009 to 2020 were assembled according to our recent report (Su et al., 2023a).

### 2.2 Features

Growth and evolution features for individual tree were organized, including cultivar (Cul), age, number of autumn flushes (NOF), DOFM, DOFE flower rate (Flr.Rate) and flower state (Flr.State). Dummy variables such as Cul.FZX, Cul.GW, Cul.HZ, Cul.NMC were created for the four cultivars. A set of odd-even year features (Year.Odd and Year.Even) was introduced to assess the interannual variability of the flower rate. According to an empirical threshold at 75% of flower rate, binary classes were categorized as Flr.Good (≥75%) and Flr.Poor (<75%).

Temperature and humidity from meteorological time-series data along flowering induction periods, as well as their evolution profiles were also generated. They have the coefficient of variation (CV), standard deviation (SD), temperature decrease per unit of time (Slneg.Temp), and temperature increase per unit of time (Slpos.Temp), the accumulations of chilling and heat calculated for different time scales. *Slpos.Temp_i_* and *Slneg.Tem*p_i_ were calculated for hourly, daily, 3-day, 5-day, 7-day, and 10-day intervals. Slpos.Temp*_t_* and *Slneg.Temp_t_* represent cumulative values of Slpos.Temp and Slneg.Temp, respectively. Chilling and heating degree calculations were used to quantify features related to accumulation (Su et al., 2023a).

A total of 229 features and 418 records were obtained through above steps. Followed by feature engineering, redundancy, low informational features were initially removed according to the *L*_sig_ criteria, which is set at 0.05, followed by eliminating features with constant values across all plants (Liu et al., 2019, 2021; Su et al., 2023a). Finally, 20, 20, 20, 19, 20, and 24 features for growth, developmental, and meteorological features were selected (**Table 1**) to build predictive models in datasets ranging from 10 days to 1 hour.

**Table 1:** Features for Flr.State predictive models of litchi.

No.	Dataset					
	Time scale=10 d	Time scale=7 d	Time scale=5 d	Time scale=3 d	Time scale=1 d	Time scale=1 h
1	Mean.Humi.Mean	Mean.Humi.Mean	Mean.Humi.Mean	Mean.Humi.Mean	Mean.Humi.Mean	Mean.Humi
2	Mean.Temp.Max	Mean.Temp.Max	Mean.Temp.Max	Mean.Temp.Max	Mean.Temp.Max	Mean.Temp
3	Mean.Temp.Min	Mean.Temp.Min	Mean.Temp.Min	Mean.Temp.Min	Mean.Temp.Min	Slpos.Temp
4	Slpos.Temp.Mean	Slpos.Temp.Mean	Slpos.Temp.Mean	Slpos.Temp.Mean	Slpos.Temp.Max	Achil.10
5	Slneg.Temp.Min	Slneg.Temp.Max	Slneg.Temp.Mean	Slneg.Temp.Mean	Slneg.Temp.Min	Achil.13
6	Achil.Min.10	Achil.Min.9	Achil.Min.8	Achil.Min.7	Achil.Min.6	Achil.6
7	Achil.Mean.14	Athrm.Min.21	Athrm.Min.21	Athrm.Min.21	Athrm.Min.22	Achil.7
8	Athrm.Mean.25	Achil.Mean.13	Achil.Mean.12	Achil.Mean.10	Achil.Mean.8	Achil.8
9	Achil.Max.19	Achil.Max.16	Achil.Max.15	Achil.Max.24	Achil.Max.10	Achil.9
10	Achil.Max.25	Achil.Max.25	Achil.Max.25	Age	Achil.Max.26	Athrm.17
11	Age	Age	Age	DOFM	Age	Athrm.21
12	DOFM	DOFM	DOFM	DFI	DOFM	Athrm.25
13	DFI	DFI	DFI	DOFE	DFI	Athrm.28
14	DOFE	DOFE	DOFE	Cul.FZX	DOFE	Athrm.31
15	Cul.FZX	Cul.FZX	Cul.FZX	Cul.GW	Cul.FZX	Age
16	Cul.GW	Cul.GW	Cul.GW	Cul.HZ	Cul.GW	DOFM
17	Cul.HZ	Cul.HZ	Cul.HZ	Cul.NMC	Cul.HZ	DFI
18	Cul.NMC	Cul.NMC	Cul.NMC	Year.Odd	Cul.NMC	DOFE
19	Year.Odd	Year.Odd	Year.Odd	Year.Even	Year.Odd	Cul.FZX
20	Year.Even	Year.Even	Year.Even		Year.Even	Cul.GW
21						Cul.HZ
22						Cul.NMC
23						Year.Odd
24						Year.Even

### 2.3 Methods

We evaluated five distinct machine learning algorithms, representing a diverse set of modeling approaches: RF, an ensemble method known for high accuracy and robustness to overfitting (Breiman, 2001); LDA, a classic linear classifier based on Bayes’ theorem (Fisher, 1936); Support Vector Machine (SVM), which finds the optimal hyperplane to separate classes (Cortes and Vapnik, 1995); Classification and Regression Trees (CART), a decision tree algorithm that partitions the feature space (Gordon et al., 1984); and K-Nearest Neighbors (KNN), a simple instance-based learner that classifies based on the majority vote of its neighbors (Fix and Hodges, 1989). RF algorithm was also used to build regression model for Flr.Rate. A preliminary comparison of their predictive performance is provided in **Supplementary Figure A1**. The training data set included data from 2009 to 2016, 2018, and 2019. Machine learning algorithms were trained with 70% of the data and verified with the remaining 30%. Random resampling and 5-fold cross-validation with 999 repetitions were used to prevent overfitting and assess model robustness. Blind testing was done on the 2017 and 2020 annual datasets. Random seeds were used for resampling and model training to ensure reproducibility. Implicit and explicit models were built with R project (version 3.5.2). The ‘caret’ package (Kuhn, 2008) was utilized to optimize the hyperparameters of machine learning algorithms.

RF and LDA shown the best performance and were selected as to build implicit (RF) and explicit (LDA) classification models. The hyperparameters for these algorithms were also optimized, typical example for Random Forest algorithm were shown in **Supplementary Figure A2**. The final predictive models enclosed 11 features, and the optimal hyperparameters for RF algorithm are 2000 decision trees with Acuracy of 0.89.

Statistical matrices to evaluate binary classification models have the area under receiver operating characteristics (ROC) curve (AUC), the confusion matrix, accuracy and Kappa values. The root mean square error (RMSE) and coefficient of determination (R^2^) were calculated to evaluate the robustness of regression models. Feature importance for predicting Flr.State was determined using RF through Gini importance-based mean decrease impurity (Gordon et al., 1984) and SHapley Additive exPlanations (SHAP) values (Lundberg et al., 2020). SHAP values, grounded in cooperative game theory, quantify each feature’s marginal contribution to a prediction by averaging its effects across all possible feature combinations, ensuring consistency and local accuracy. For each sample, a feature’s SHAP value represents its additive impact on the model’s prediction deviation from the baseline (population mean). Global feature importance was assessed by averaging absolute SHAP values across all samples, while SHAP summary and dependence plots visually decoded the directional, magnitude, and interactive effects of features (**Figures 2**, **3**). The Workflow to predict the litchi flower rate was presented in **Figure 4**.

**Figure 2:**
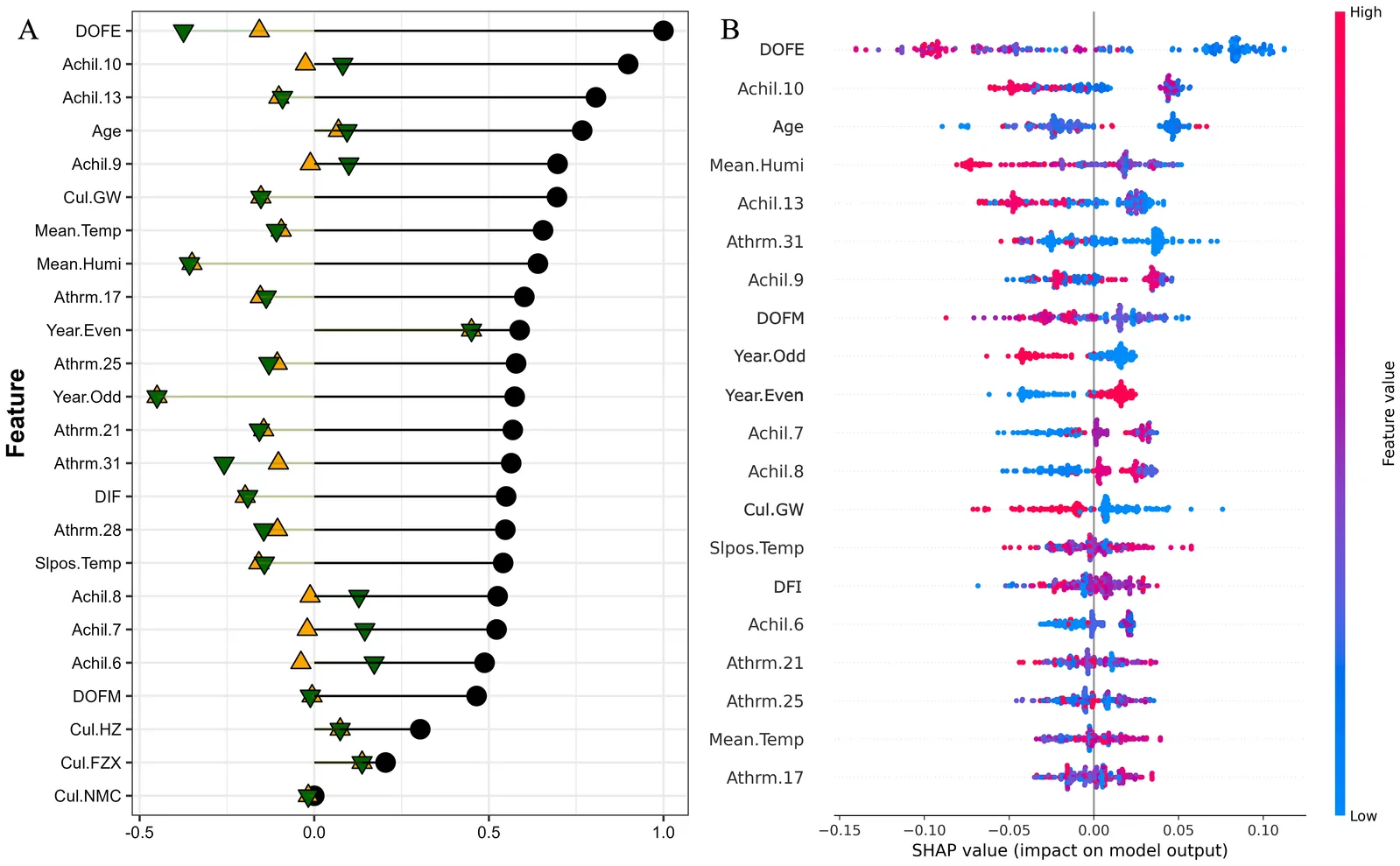
Feature importance of the RF model in 1-hour timescale data. **(A)** ranking of feature importance (black circle) in 1-hour timescale data set, Pearson (orange triangle) and Spearman (dark green triangle) correlation coefficients against Flr.State based on mean decrease accuracy of RF classifier. **(B)** SHAP summary plots of the RF model, each sample is colored by its corresponding feature value with higher values shown in pink and lower values in blue.

**Figure 3:**
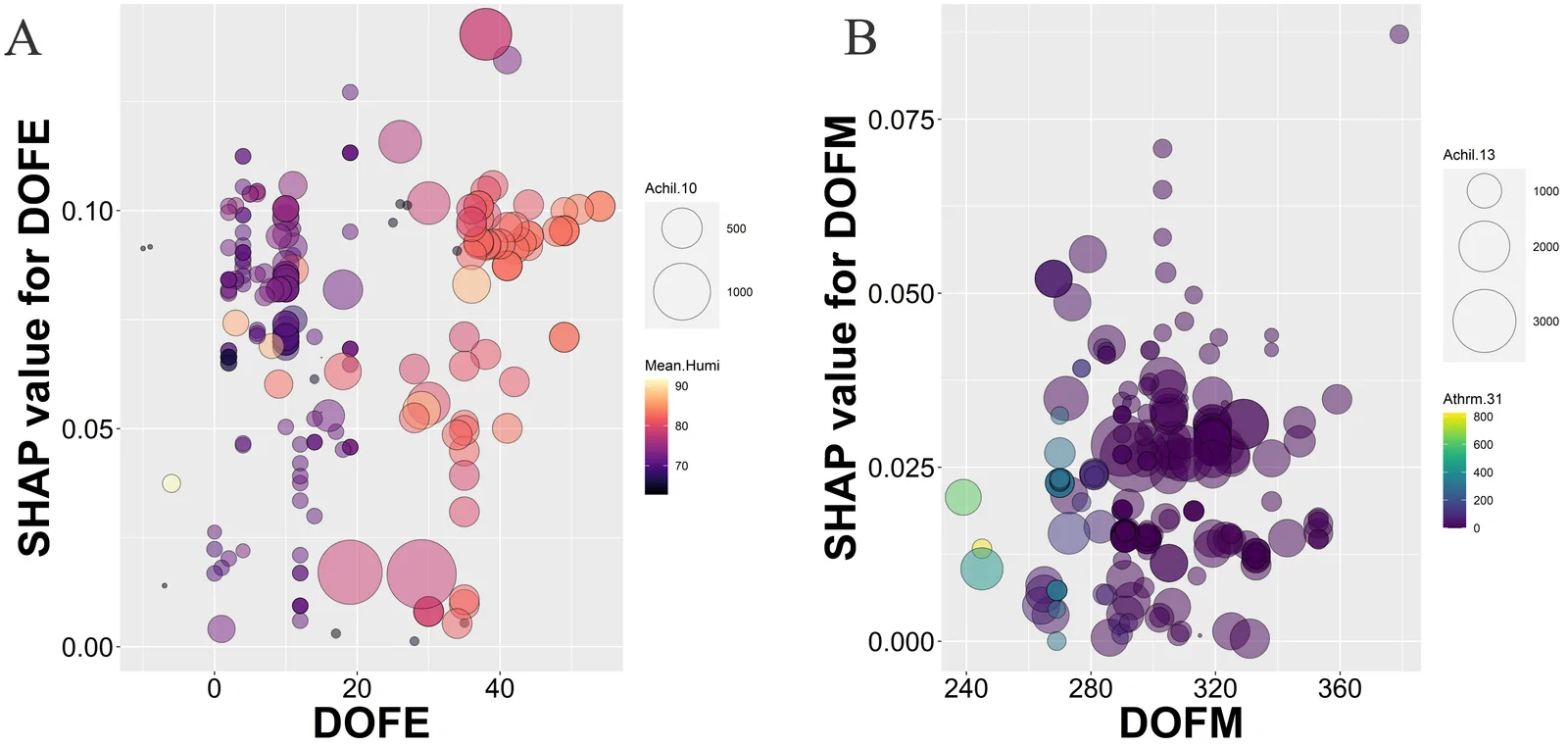
Dependency plot of SHAP value and variables. **(A)** reveals the impact of DOFE on model output and the interactive effects between Achill.10, Mean.Humi and DOFE. **(B)** reveals the impact of DOFM on model output and the interactive effects between Achill.13, Athrm.31 and DOFM.

**Figure 4:**
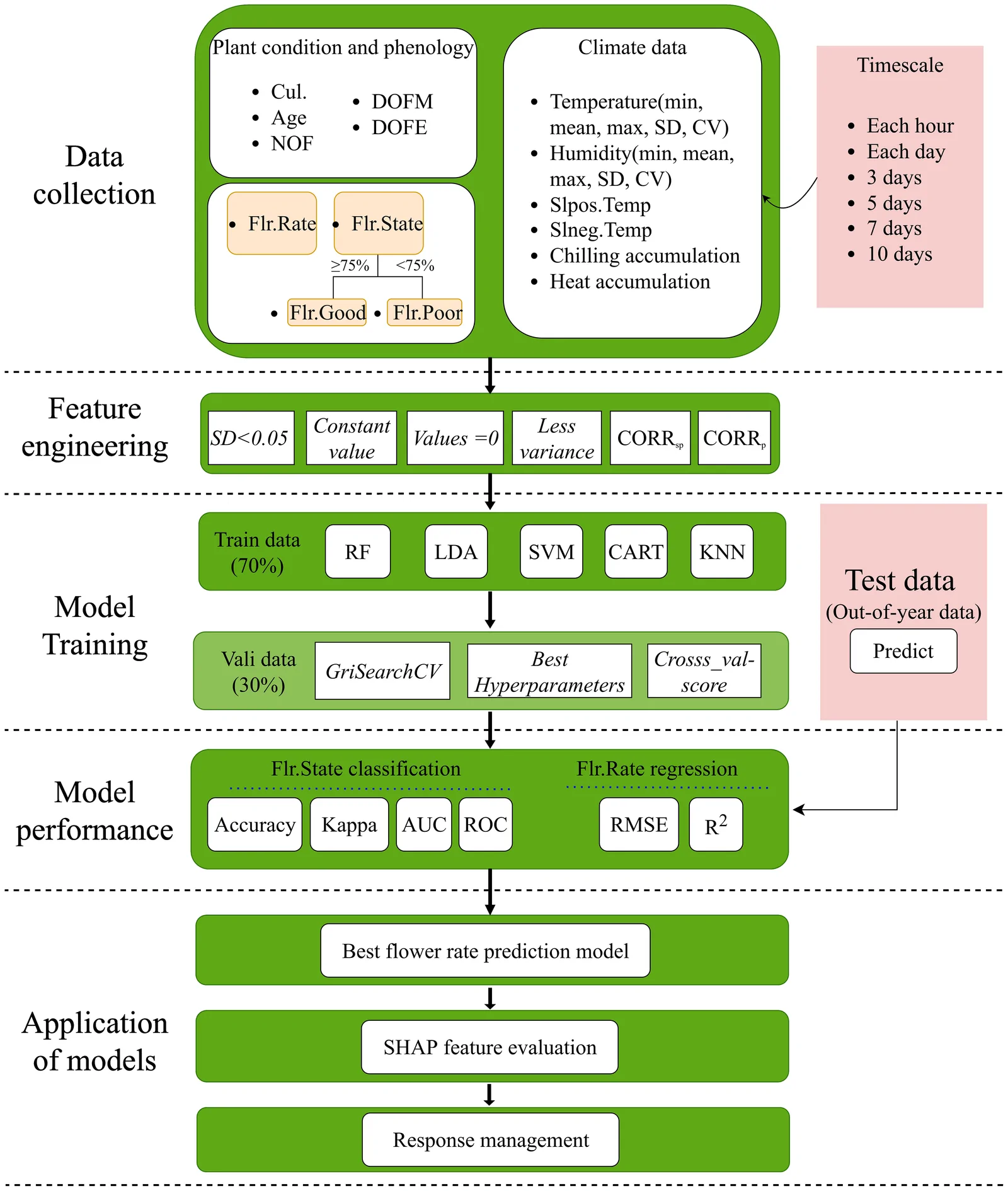
Workflow to predict the litchi flower rate base on machine learning.

## 3 Results

### 3.1 Distribution of flower rates and features

‘FZX’ and ‘HZ’ had a high flower rate close to 90% in most years, while ‘GW’ and ‘NMC’ had a bimodal distribution with peaks around 75% and 25% (**Figure 5**). These distributions of flower rates are significantly biased from Gaussian distribution, may fluctuate in a range from 0-100%, empirically shown strong dependence on many factors. It suggested appreciate response management of individual trees is critical to improve the harvest through enhanced flower rate. Our previous research indicated that DOFM, DOFE, DFI, daily mean temperature, and humidity during flower induction in litchi (Su et al., 2023a) are top-ranked important factors. The distribution in DOFM for four cultivars closely resemble a Gaussian with median DOFM ranging from 292 to 304 DOY. The DOFE of ‘FZX’ was earlier than the other three, occurring between -10 and 38 DOY while ‘NMC’ initiated flowering later, falling between 1 and 55 DOY. ‘FZX’ also demonstrated the most extensive time span for flower induction, ranging from 38 to 148 days. The DFI of ‘HZ’, ‘GW’, and ‘NMC’ had a consistent pattern with three stages of daily temperature change. Peaks in temperature occurred around 23 °C -28 °C and 15 °C, with high and low temperature accumulation during different stages. Relative humidity typically ranged from 70% to 90%.

**Figure 5:**
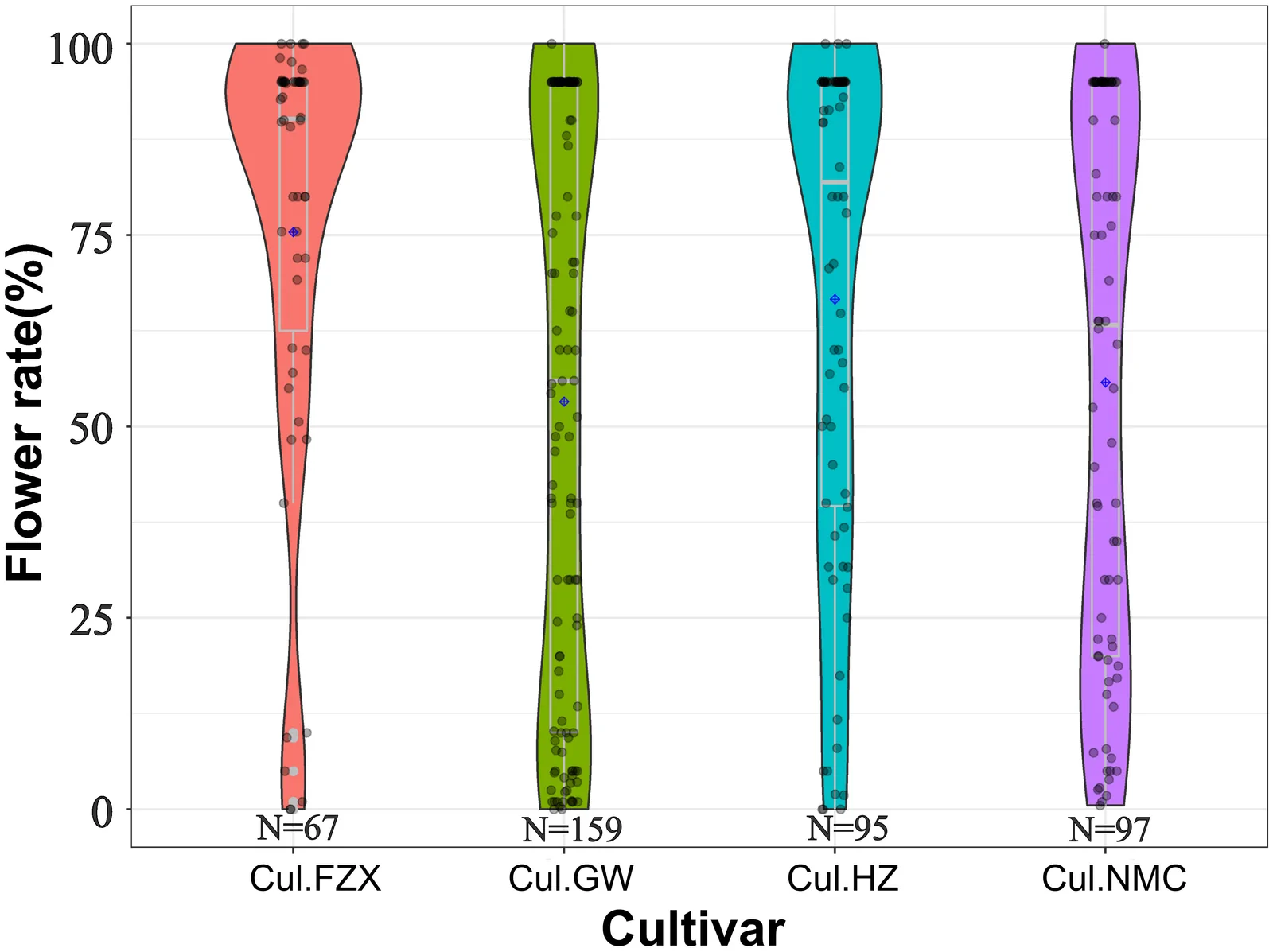
The distribution of flower rates for the four types of litchi cultivars. N is the number of trees in each cultivar.

Beyond these distributions, their linear and monotonous correlations were also evaluated using Pearson (CORR_p_) and Spearman (CORR_sp_) correlation coefficients. The correlation matrix was shown in **Supplementary Figure A3**. Strong negative correlation for single feature with Flr.State and Flr.Rate have DOFE. It’s reasonable that short duration of flower induction, delayed of floral white millet emergence often result in low flower rate. Other features like Age and Achil.Min.8 had low correlations with Flr.State and Flr.Rate. The results showed that flower was influenced by multiple factors, and the correlation between hot and cold units and flower rate is low. To address this, we used machine learning algorithms to analyze the relationship.

### 3.2 Predictive models

An initial evaluation was carried out to compare the predictive performance for six algorithms including RF, LDA, SVM, CART, and KNN. As shown in **Supplementary Figure A1**, RF, LDA, and SVM algorithms exhibited an accuracy higher than 0.8 and a Kappa value of around 0.60, suggesting these models are reliable. For a consideration of implicit and explicit predictive models, RF and LDA predictive models in the classification of Flr.State were selected for further analysis.

**Figure 6** displayed the predictive performance of classification and regression models. ROC curves for training, validation, and two blind tests (2017 and 2020) using RF (**Figure 6A**) and LDA (**Figure 6B**) for Flr.State were shown, with statistical metrics in **Table 2**. Predicted vs. observed Flr.Rate plots for training and validation data were in **Figure 6C**, and for blind test data (2017 and 2020) in **Figure 6D**, with metrics summarized in **Table 3**. The model’s predictive ability was tested using the validation set, with the RF model outperforming the LDA model in Sensitivity, Specificity, Kappa, Accuracy, and AUC (**Table 2**). Both classification models also performed well in blind tests from 2017 and 2020, with AUC values above 0.60 (**Table 4**; **Figure 6**), indicating good internal and external prediction capabilities.

**Figure 6:**
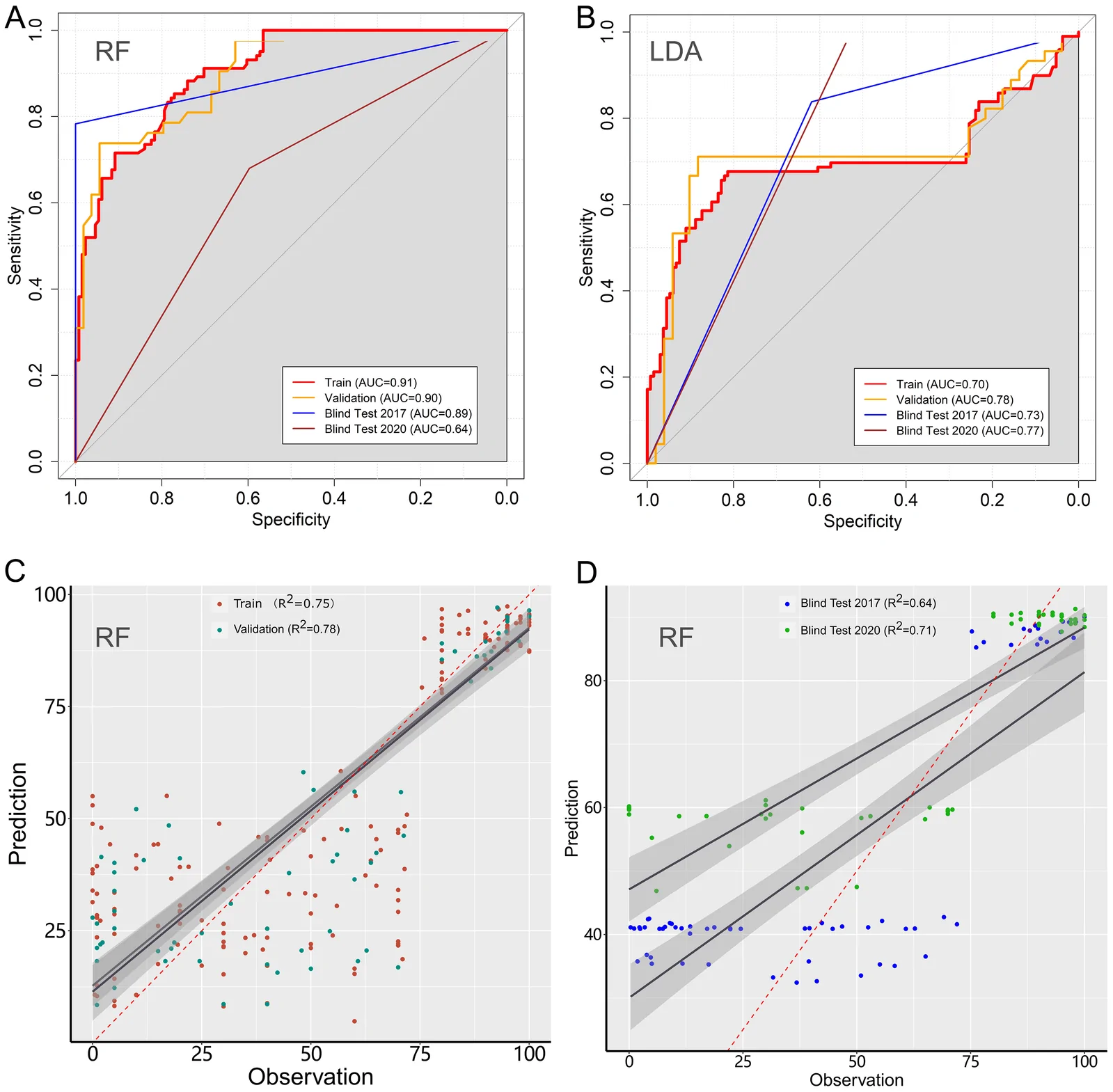
ROC curves for Flr.State predictions using RF **(A)** and LDA **(B)** in training, validation, and blind tests (2017, 2020). Predicted vs observed Flr.Rate plots using RF for training and validation **(C)**, and blind tests (2017, 2020) **(D)**. RF, Random Forest; LDA, Linear Discriminant Analysis; AUC, Area Under the Curve.

**Table 2:** The prediction performance evaluation of classification models on the validation dataset (71 records).

Model	Dataset	No. of features	Records	Validation				
				Sensitivity	Specificity	Kappa	Accuracy	AUC (95% CI)
RF model								
10 d	Time scale =10 days	20	326	0.84	1.00	0.79	0.90	0.89 (0.81-0.96)
7 d	Time scale =7 days	20	326	0.71	1.00	0.63	0.81	0.82 (0.75-0.90)
5 d	Time scale =5 days	20	326	0.89	0.94	0.83	0.91	0.90 (0.85-0.98)
3 d	Time scale =3 days	19	326	0.79	0.84	0.62	0.81	0.81 (0.72-0.90)
1 d	Time scale =1 day	20	326	0.81	0.93	0.71	0.86	0.85 (0.77-0.93)
1 h	Time scale =1 hour	24	326	0.81	0.89	0.68	0.84	0.83 (0.75-0.93)
LDA model								
10 d	Time scale =10 days	20	326	0.83	0.96	0.77	0.88	0.88 (0.81-0.96)
7 d	Time scale =7 days	20	326	0.73	0.96	0.66	0.83	0.83 (0.72-0.91)
5 d	Time scale =5 days	20	326	0.74	0.87	0.55	0.78	0.78 (0.69-0.88)
3 d	Time scale =3 days	19	326	0.76	0.87	0.62	0.81	0.81 (0.72-0.91)
1 d	Time scale =1 day	20	326	0.81	0.85	0.64	0.83	0.82 (0.73-0.91)
1 h	Time scale =1 hour	24	326	0.78	0.88	0.65	0.83	0.83 (0.74-0.92)

**Table 3:** The prediction performance evaluation of the RF regression models.

Dataset	Training			Validation			Blind test (2017)			Blind test (2020)		
	RMSE	R^2^	MAE	RMSE	R^2^	MAE	RMSE	R^2^	MAE	RMSE	R^2^	MAE
Time scale =10 days	17.18	0.76	11.24	16.85	0.77	10.48	3.78	0.57	18.74	20.74	0.71	14.44
Time scale =7 days	17.27	0.76	11.30	16.82	0.77	10.60	25.72	0.56	19.93	22.47	0.72	15.19
Time scale =5 days	18.12	0.75	11.68	15.80	0.78	10.43	23.77	0.64	19.30	24.97	0.71	16.71
Time scale =3 days	17.07	0.77	11.18	16.95	0.77	10.64	23.26	0.56	18.47	20.25	0.73	13.53
Time scale =1 day	17.21	0.76	11.26	17.09	0.76	10.91	24.65	0.55	19.33	18.95	0.71	13.09
Time scale =1 hour	17.20	0.76	11.27	17.26	0.76	10.94	24.75	0.53	19.54	21.83	0.70	14.91

**Table 4:** The prediction performance evaluation of classification models on the blind test dataset from 2017 (89 records) and 2020 (92 records).

Model	Dataset	Blind test (2017)					Blind test (2020)				
		Sensitivity	Specificity	Kappa	Accuracy	AUC (95% CI)	Sensitivity	Specificity	Kappa	Accuracy	AUC (95% CI)
RF	Time scale =10 days	0.29	1.00	0.38	0.81	0.90 (0.85-0.94)	1.00	0.00	0.00	0.52	–
	Time scale =7 days	0.33	0.86	0.21	0.72	0.62 (0.49-0.76)	1.00	0.00	0.00	0.52	–
	Time scale =5 days	0.25	1.00	0.33	0.80	0.89 (0.85-0.94)	0.83	0.39	0.22	0.62	0.64 (0.53-0.75)
	Time scale =3 days	0.33	1.00	0.42	0.82	0.90 (0.86-0.95)	1.00	0.0	0.00	0.52	–
	Time scale =1 day	0.33	1.00	0.42	0.82	0.90 (0.86-0.95)	1.00	0.0	0.00	0.52	–
	Time scale =1 hour	0.29	1.00	0.38	0.81	0.90 (0.85-0.94)	1.00	0.02	0.02	0.53	0.76 (0.43-0.64)
LDA	Time scale =10 days	1.00	0.00	0.00	0.27	–	1.00	0.00	0.00	0.52	–
	Time scale =7 days	0.75	0.00	-0.14	0.20	0.11 (0.06-0.15)	1.00	0.02	0.02	0.53	0.76 (0.43-0.64)
	Time scale =5 days	0.54	0.88	0.44	0.79	0.73 (0.61-0.84)	1.00	0.02	0.02	0.53	0.77 (0.43-0.64)
	Time scale =3 days	0.67	0.42	0.06	0.48	0.53 (0.44-0.63)	1.00	0.00	0.00	0.52	–
	Time scale =1 day	1.00	0.00	0.00	0.27	–	1.00	0.00	0.00	0.52	–
	Time scale =1 hour	0.08	0.92	0.01	0.70	0.51 (0.32-0.70)	1.00	0.00	0.00	0.52	–

The inclusion of accumulated estimation signals for temperature and humidity enhances the predictive capabilities of the model. A comprehensive analysis was conducted to compare the predictive performance and reliability of various time scale datasets, as shown in **Tables 2**, **4**. The parameters of each model were fine-tuned to achieve optimal results.

The 1-hour timescale dataset demonstrated the highest accuracy in the Random Forest (RF) model, with AUC values of 0.83, 0.90, and 0.76 for the Validation, Blind test (2017), and Blind test (2020) datasets, respectively. The LDA model with a 5-day timescale had the highest accuracy, with AUC values of 0.78, 0.73, and 0.77 for Validation, Blind test (2017), and Blind test (2020). Excluding the blind test sets from training and validation showed the accuracy and stability of the models. Overall, the RF model using a 1-hour timescale and the LDA model using a 5-day timescale provided the most accurate predictions. Here is the complete equation for predicting the Flr.Good from the LDA model:

*Flr.Good* =–(0.56×Mean.Humi.mean) – (1.65×Mean.Temp.max) + (4.79×Mean.Temp.min) + (2.27×Slpos.Temp.mean) + (1.51×Slneg.Temp.mean) + (2.43×Achil.min.8) + (3.54×Athrm.min.21) + (10.51×Achil.mean.12) + (5.53×Achil.max.15) + (5.85×Achil.max.25) – (0.97×Age) – (2.26×DOFM) – (10.14×DFI) + (0.18×DOFE) – (0.46×Cul.FZX) + (0.30×Cul.GW) + (0.10×Cul.HZ) – (0.16×Cul.NMC) + (1.05×Year.Odd) – (1.05×Year.Even) (1).

Used the same datasets and optimal number of features, RF was also applied to construct regression models. Predicted performance indices were summarized in **Table 3**. The R^2^ reached 0.78, 0.64 and 0.71 for Validation, Blind test (2017), and Blind test (2020) datasets, respectively.

### 3.3 Feature importance and correlation evaluation

To determine the key factors influencing Flr.State, we analyzed feature importance using %IncMSE, CORR_p_, CORR_sp_, and SHAP values. Features with the highest impact were ranked accordingly (**Figure 2**) CORR_p_ and CORR_sp_ showed similar trends in assessing feature information on Flr.State.

DOFE was the top feature in both RF (**Figure 2A**), indicating its strong interpretability for Flr.State. Other important features included Achil.10, Age, Mean.Humi, Achil.13, Achil.9, and Year.Even. %IncMSE and mean SHAP values identify important features, while CORR_p_, CORR_sp_, and SHAP summary plots show how a feature impacts the prediction. DOFE and Mean.Humi were negatively correlated with Flr.State, indicating that later floral white millet emergence and higher humidity led to a lower flower rate. Achil.10, Achil.9, Achil.8, and Achil.7 were positively correlated with Flr.State, showing that higher accumulation of chilling below certain temperatures resulted in a higher flower rate (**Figure 2**).

Further analysis of the relationship between Flr.State and important variables was done by plotting feature values against their SHAP values (**Figure 3**). Smaller DOFE values are associated with higher SHAP values, indicating a negative correlation with Flr.State. Plants with early floral white millet emergence are more likely to have a high flower rate. The date of floral white millet emergence determines Flr.State. Postponing the date of floral white millet emergence after 15 DOY could lead to a decline in flower rate. **Figure 3A** illustrated that the high value of the Achil.10 feature was predominantly observed in regions characterized by a low variable value of DOFE (located on the right side of the figure). Conversely, the low value of the Mean.Humi feature was concentrated in areas with a small variable value of DOFE (located on the left side of the figure). This suggested that plants exhibiting early floral white millet emergence were likely to receive higher chilling accumulation below 10 °C and encounter increased drought conditions, ultimately leading to a higher flower rate. It is important to note that the maturation of the last shoots in autumn serves as a physiological prerequisite for litchi plants to transition into the floral induction stage. DOFM was a key factor influencing flowering phenology (Su et al., 2021), with higher temperatures during this period. The relationship between SHAP values and DOFM, Achill.13, and Athrm.31 was explored (**Figure 3B**). DOFM had the most significant impact on the model between 280 and 304 DOY, decreasing in importance as the date was postponed. Plants with early last autumn flush maturation in the area with low DOFM values would experience less chilling accumulation between 13-10 °C and heat accumulation below 31 °C after 280 DOY.

## 4 Discussion

### 4.1 Performance of the models

Machine learning techniques can combine multiple features and their dependencies to outperform traditional statistical methods (Topçuoğlu et al., 2020). Multivariate analysis is more effective than individual analysis in predicting Flr.State in litchi (Fortino et al., 2020). Therefore, we developed two machine learning models to predict Flr.State under various microclimate features collected from 2009-2020.

This methodological choice is grounded not merely in the empirical popularity of machine learning, but in the fundamental mismatch between the assumptions of conventional regression and the biological reality of litchi flowering. Conventional regression models, whether linear or logistic, presume additive effects and often require pre-specification of interaction terms, yet the floral induction process is governed by threshold responses (e.g., chilling requirements below specific temperatures), non-linear interactions between environmental and phenological factors, and context-dependent effects that vary across cultivars and years. C Moreover, as shown in **Supplementary Figure A3**, the weak pairwise correlations between individual features and Flr.State indicate that no single predictor or simple linear combination of predictors can adequately capture flowering outcomes—a diagnostic signature that the predictive signal resides in higher-order interactions rather than in additive marginal effects. Given that our primary objective is not to test the statistical significance of individual coefficients but to identify key drivers for actionable management decisions, the ML framework is inherently better aligned with both the scientific question and the data structure. We therefore consider the comparison with conventional regression as a methodological choice of model families rather than a performance competition; the RF and LDA models presented here represent two complementary approaches to classification (non-linear ensemble versus linear discriminant), and their relative strengths are evaluated within this biologically informed modeling framework.

In order to identify potentially significant indicators, it is imperative to assess the value of each feature in an impartial manner, considering both its statistical significance and its predictive accuracy for independent samples. Following feature engineering, the limited linear correlation observed between individual cumulative hot and cold units and Flr.State indicated the absence of redundancy and noise in the features, suggesting that Flr.State was not merely a composite of these variables (**Supplementary Figure A3**). Training models using small datasets may create overfitting models that are too sensitive to outliers and noise. In this study, we tested the separability of two categories (Flr.good and Flr.poor) using RF algorithms and LDA algorithms and various features. The algorithm successfully distinguished between the categories and provided accurate predictions of Flr.State (**Supplementary Figure A1**). Random Forest effectively handles high-dimensional datasets with multiple features by creating decision tree collections. It prevents overfitting with out-of-bag estimates and performs well on imbalanced datasets (Breiman, 2001). LDA is a reliable classifier for multi-class problems and converges to the optimal classifier with large sample sizes and small dimension (Fisher, 1936). In our work, LDA provided a convenient method to compare the posterior parameter distributions associated with each outcome to determine the parameter combination that best distinguishes Flr.State.

The incorporation of a validation strategy within the machine learning pipeline is crucial for assessing the generalizability of a model. One widely used technique for validation is *k*-fold cross-validation, which involves iteratively selecting subsets of data for training and validation to ensure the stability of the model when presented with new data. As indicated in **Tables 2** and **4**, the AUC was 0.83 for RF in the 1-hour timescale dataset and 0.78 for LDA in the 5-day timescale dataset for the validation dataset for classification models. And the R^2^ spanned 0.76 to 0.78 for the validation dataset for regression models.

To enhance the generalizability of our approach and mitigate potential biases in the samples, we systematically utilized independent database samples for training the machine learning model and conducted testing using two additional independent samples over a two-year period. The AUC for RF in the 1-hour timescale dataset was 0.90, with values of 0.76 observed for the blind tests conducted in 2017 and 2020, respectively. In the context of the 5-day timescale dataset, the AUC values for LDA were determined to be 0.73 and 0.77 for blind tests conducted in 2017 and 2020, respectively, as indicated in **Tables 2** and **4**. The model exhibited a high level of generalizability. The R^2^ values for RF was determined to be 0.64 and 0.71 for blind tests conducted in 2017 and 2020, respectively. further demonstrating the robustness of the prediction model.

Recent research has highlighted the influence of varying time scales of data on predictive models, such as the impact of different time scales of mean flowering onset dates on seasonal hybrid models (Morris and Lutscher, 2021). Additionally, our recent investigation suggested that short-term fluctuations in weather conditions can impact the duration of floral induction in litchi plants (Su et al., 2023a). The objective of this study was to examine the potential influence of weather fluctuations at varying temporal intervals on the flower rate. More specifically, the study assessed the efficacy and reliability of Flr.State prediction models across different time scales. Initially, temperature and humidity data on a 5-day interval were utilized to construct the predictive model. Subsequently, prediction models for various time scales were developed using identical screening features and tuning strategies. Finally, the study conducted an analysis of the robustness and accuracy of the models, with comparisons made to determine the optimal time scales and predictive models. Both RF and LDA models demonstrated strong performance on validation data within the time range of 1 hour to 10 days.

Results indicated that accuracy and AUC improved with extended time scales of 10 days or shortened to 1-hour time scales (**Table 2**). The accuracy of the prediction model decreased when applied to blind test datasets. The RF model was more robust at a 1-hour time scale, while the LDA model was more robust at a 1-day time scale. The RF model had better generalization performance with accuracy ranging from 0.52 to 0.81 compared to the LDA model with accuracy ranging from 0.20 to 0.79 (**Table 4**). The RF model based on a 1-hour time scale had the highest accuracy and robustness, suggesting that extreme hourly values may significantly impact Flr.State. In the blind test dataset, the model demonstrated strong performance in differentiating between Flr.Good and Flr.Poor samples, as indicated by high AUC values. However, the model exhibited a tendency towards predicting Flr.Good samples (i.e., high Sensitivity), potentially leading to a decrease in overall accuracy.

### 4.2 Expertized interpretation for importance features

The variability of litchi flower rates is influenced by a complex interplay of meteorological factors, plant growth conditions, and phenological stages conducive to floral induction. Identifying the primary limiting factors and disentangling their individual contributions from such intricate interactions has long posed challenges. To address these knowledge gaps, this study developed a Flr.State machine learning model integrating key factors and advanced interpretability techniques. The RF algorithm nonlinearly evaluates feature importance, complementing traditional correlation coefficients that capture linear and monotonic relationships. The SHAP summary charts effectively visualize feature contributions, importance rankings, and directionality of effects. Beyond meteorological predictors like chilling and heat accumulation, our model uncovers the DOFM and NOF—as well as their physiological underpinnings on litchi flowering potential.

Consistent with established horticultural principles, the model highlights that fully matured autumn shoots are an obligate prerequisite for successful floral induction (Menzel, 1983; O’Hare, 2002). Biochemically, lignified mature canes serve as central reservoirs for photosynthates (e.g., starch and soluble sugars). Sufficient carbohydrate accumulation elevates tissue C/N ratios, generating the metabolic signal that triggers and sustains floral bud differentiation (Yuan et al., 2009). Collectively, the model’s attribution of high importance to DOFM reinforces a fundamental tenet: synchronized autumn flush emergence and timely late-season shoot maturation are not merely descriptive phenological milestones, but critical physiological preconditions enabling efficient sensing and response to flower-promoting chilling signals during floral induction.

DOFE, or the emergence of ‘white millet’ in litchi, was found to strongly influence Flr.State. Previous research also found a significant negative correlation between litchi flower rate and DOFE (Su et al., 2023b). Late emergence of ‘white millet’ in litchi can result in poor flowering due to various factors such as susceptibility to reverse to leaf buds, shortened development time of inflorescence (Menzel and Simpson, 1995), and poor development of late blooming flowers in prolonged rainy weather (Menzel et al., 1989). Thus, timely promotion of “white millet” after floral induction can help litchi buds develop into inflorescences during early spring.

The relationship between chilling and heat accumulation on Flr.State and DFI is complex (Su et al., 2023a), with various studies attempting to predict spring phenology (Dai et al., 2019; Janosi et al., 2020) in temperate woody species. The findings of these studies may not be generalizable to litchi trees in tropical and subtropical regions. Previous research has indicated that the temperature range necessary for litchi floral induction is between 5 and 14 °C (Groff, 1943). Exposure to temperatures exceeding 20 °C for more than 8 hours per day has been shown to have a detrimental effect on flowering accumulation in litchi (Menzel and Simpson, 1995). Our model elucidates the positive effects of Achil.10, Achil.9, Achil.8, and Achil.7 on Flr.State, alongside the negative impacts of Achil.13 and Athrm.31. Furthermore, Mean.Humi is identified as a significant negative driver, corroborating prior studies indicating that drought synergizes with cold stress to enhance flowering (Menzel et al., 1989; Carr and Menzel, 2014; Shen et al., 2016). Notably, interannual variables (Year.Even and Year.Odd) exhibit importance >0.5, underscoring substantial year-to-year fluctuations.

SHAP plots excel in revealing nuanced trends within subsets. While DOFM ranks modestly in global importance (RF model), it emerges as the second most influential local driver of reduced Flr.State in specific data subsets—surpassed only by DOFE (**Figure 2B**). This pattern would be obscured by global feature importance plots alone (**Figure 2A**), highlighting the necessity of local explainability techniques to capture context-dependent effects. Thus, our interdisciplinary approach not only validates traditional horticultural knowledge but also uncovers actionable physiological-meteorological interactions for targeted cultivation strategies.

### 4.3 Limitations and potential improvements

The formation of litchi flowers is jointly determined by environmental-climatic factors and internal nutrition levels (Prasad et al., 2016; Ye and Sakai, 2016). While technical constraints limited individual plant nutrient monitoring, this study explored odd-even years as a proxy for annual variation, with Year.Odd/Even ranking significantly. However, integrating biochemical indicators—such as leaf C/N ratios, phytohormone (e.g., gibberellins, ABA, cytokinins) levels, and autumn shoot carbohydrates—would enhance model efficacy. These traits mechanistically link to prioritized morphological/phenological features (e.g., DOFM reflecting shoot maturation) but physiological differences (e.g., carbohydrate reserves, hormone profiles) can cause divergent flowering outcomes despite similar environmental conditions. For instance, Mean.Humi’s negative effect on Flr.State (**Figure 2**) likely involves humidity-altered carbohydrate metabolism and hormone balance (Shen et al., 2016), yet current models indirectly infer this. Biochemical data would thus improve predictions and disentangle environmental effects. Recent advancements in portable near-infrared spectroscopy and hyperspectral imaging (Chlingaryan et al., 2018; Zhu et al., 2020; Tang et al., 2023) now enable feasible *in situ* estimation of these parameters.

The feature importance analysis revealed that Achi.10 significant impacts on Flr.State. However, regional adaptation—e.g., in Hainan Province where monthly temperatures exceed 10 °C—requires substituting datasets to reflect local conditions. Classification and aggregation level variations can alter feature weights, potentially elevating or diminishing certain influences. While these models demonstrate predictive precision, validating their generalizability across cultivars, regions, and seasons is imperative. Systematic field validations in diverse environments are thus prioritized to assess model robustness under fluctuating climatic and phenological conditions.

## 5 Conclusions

In this work, robust predictive model for the flower state of four litchi cultivars were constructed. The accuracy to predict trees with higher than 75% flower rate reached 0.90. The exploration of important features revealed that flower rate of litchi was sensitive to the date of floral white millet emergence, the accumulation of chilling hours below 9, 10 and 13 °C in the process of flower induction, the accumulation of heat hours above 31 °C in the process of flower induction, etc. The findings of our study demonstrate that both a classification models and regression models exhibit predictive accuracy. Integration of explicit and implicit algorithms can enhance the precision of prediction models by mitigating uncertainty. These models consider various factors including cultivar, age, interannual variation, temperature, humidity changes, and temporal data fluctuations. To identify the primary determinant of Flr.State, the RF model can be augmented with the SHAP method. The refinement of models can be enhanced by incorporating data pertaining to the internal state of litchi and data from diverse geographical regions. These insights will assist growers in devising more precise management tactics to enhance the flower rate of litchi. For instance, growers can manipulate the maturation of flush to facilitate floral induction in accordance with meteorological variables of significant importance, and subsequently stimulate the timely emergence of “white millet” through irrigation, pruning, and foliar fertilization (**Figure 7**).

**Figure 7:**
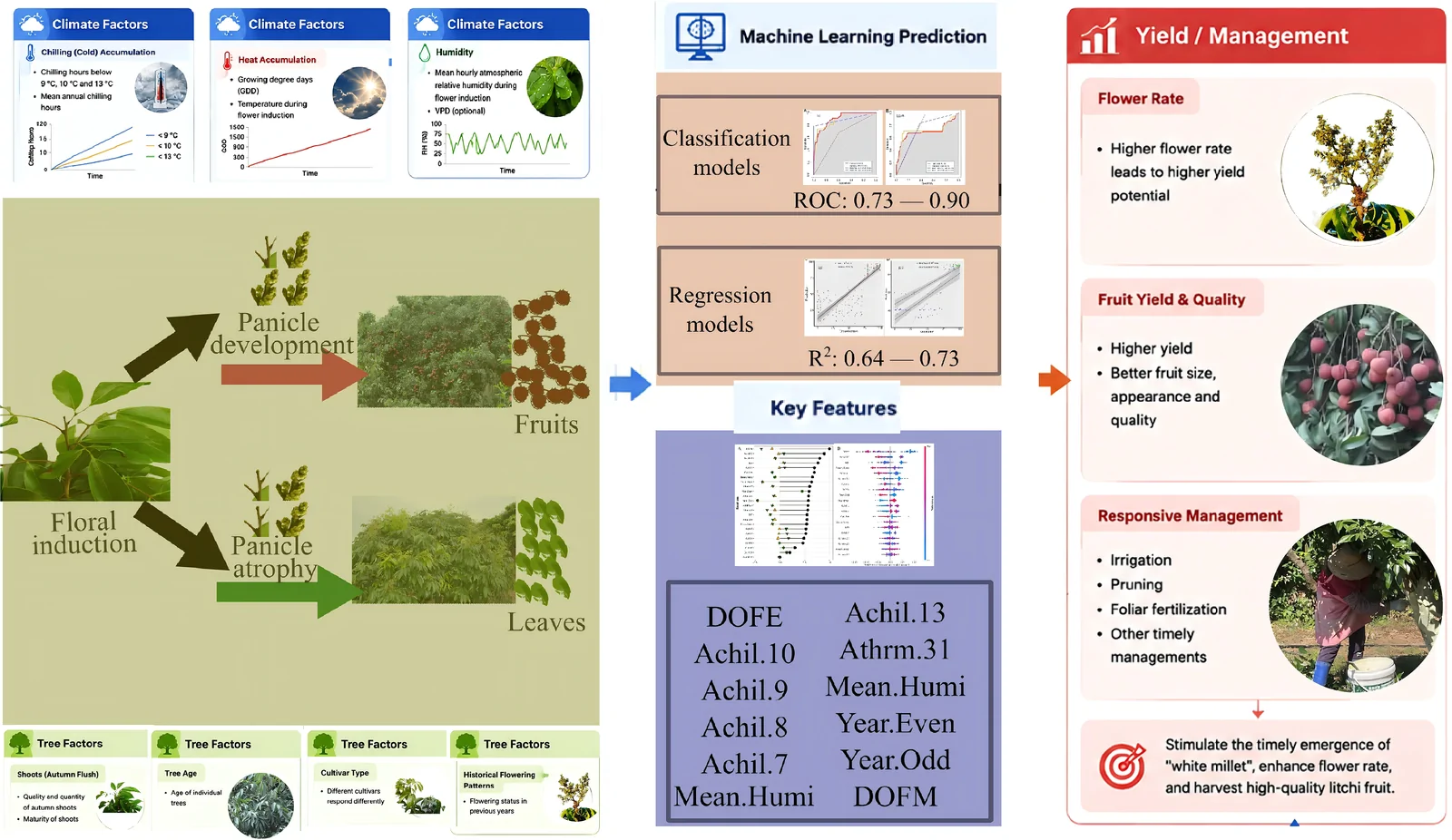
Workflow of machine learning-based litchi flowering prediction and precision orchard management.

## Data Availability

The raw data supporting the conclusions of this article will be made available by the authors, without undue reservation.

## Glossary

%IncMSE: Per cent increase in mean squared error
Achil.(5-35): The accumulation of chilling hours beneath the critical air temperatures indicated in parentheses in the process of flower induction
Achil.Max.(5-35): The accumulation of chilling per day about the maximum air temperature beneath the critical air temperatures indicated in parentheses in the process of flower induction
Achil.Mean.(5-35): The accumulation of chilling per day about the mean air temperature beneath the critical air temperatures indicated in parentheses in the process of flower induction
Achil.Min.(5-35): The accumulation of chilling per day about the minimum air temperature beneath the critical air temperatures indicated in parentheses in the process of flower induction
ANOVA: Analysis of variance
Athrm.(5-35): The accumulation of heat hours above the critical air temperatures in parentheses in the process of flower induction
Athrm.Max.(5-35): The accumulation of heat per day about the maximum air temperature above the critical temperatures in parentheses in the process of flower induction
Athrm.Mean.(5-35): The accumulation of heat per day about the mean air temperature above the critical temperatures in parentheses in the process of flower induction
Athrm.Min.(5-35): The accumulation of heat per day about the minimum air temperature above the critical temperatures in parentheses in the process of flower induction
AUC: The area under the receiver operating characteristic curve
CART: Classification and Regression Trees algorithm
CORR_p_: Pearson correlation coefficient
CORR_Sp_: Spearman correlation coefficient
Cul: Cultivar
Cul.FZX: Dummy variables about cultivar 'feizixiao'
Cul.GW: Dummy variables about cultivar 'guiwei'
Cul.HZ: Dummy variables about cultivar 'huaizhi'
Cul.NMC: Dummy variables about cultivar 'nuomici'
CV: Coefficient variation
d: Day
DFI: The duration of flower induction
DOFE: The date of floral white millet emergence
DOFM: The date of the last autumn flush maturation
DOY: Day of year
Flr.Good: Flowrering rate ≥75%
Flr.Poor: Flowrering rate <75%
Flr.Rate: Flower rate, Flr.State, Flowrer state
FN: False negative
FP: False positive
FZX: 'Feizixiao'
GW: 'Guiwei'
h: Hour
Humi.Mean: Mean atmospheric relative humidity
HZ: 'Huaizhi'
KNN: K-NearestNeighbor algorithm
LDA: Linear discriminant analysis algorithm
MAE: Mean absolute error
Mean.Humi: The mean atmospheric relative humidity per hour in the process of flower induction
Mean.Humi.Max: The mean of max atmospheric relative humidity per day in the process of flower induction
Mean.Humi.Mean: The mean of mean atmospheric relative humidity per day in the process of flower induction
Mean.Humi.Min: The mean of minimum atmospheric relative humidity per day in the process of flower induction
Mean.Temp: The mean temperature per hour in the process of flower induction
Mean.Temp.Max: The mean of max temperature per day in the process of flower induction
Mean.Temp.Mean: The mean of mean temperature per day in the process of flower induction
Mean.Temp.Min: The mean of minimum temperature per day in the process of flower induction
NMC: 'Nuomici'
NOF: Number of autumn flushes
RF: Random forest algorithm
RMSE: Root mean square error
ROC: Receiver operating characteristic curve
R_p_^2^: The coefficient of determination
SD: Standard deviation
SE: Standard error
SHAP: SHapley Additive exPlanations
Slneg.Temp: The sum of the rate of temperature drop per hour in the process of flower induction
Slneg.Temp.Max: The sum of the rate of maximum temperature drop per day in the process of flower induction
Slneg.Temp.Mean: The sum of the rate of mean temperature drop per day in the process of flower induction
Slneg.Temp.Min: The sum of the rate of minimum temperature drop per day in the process of flower induction
Slpos.Temp: The sum of the rate of temperature rise per hour in the process of flower induction
Slpos.Temp.Max: The sum of the rate of max temperature rise per day in the process of flower induction
Slpos.Temp.Mean: The sum of the rate of mean temperature rise per day in the process of flower induction
Slpos.Temp.Min: The sum of the rate of min temperature rise per day in the process of flower induction
SVM: Support Vector Machine algorithm,
Temp.Mean: Mean temperature
TN: True negative
TP: True positive
Year.Even: Even year
Year.Odd: Odd year
