# Supplementary material for: Understanding the flower rate of litchi (Litchi chinensis Sonn.) through machine learning predictive models
Source: Front Plant Sci. 2026 Oct 27;17:1865699. doi: 10.3389/fpls.2026.1865699 (PMC13748220; doi:10.3389/fpls.2026.1865699)
Supplement: Supplementary file 1 [file SupplementaryFile1.docx]

**Predicting flower induction of litchi (*Litchi chinensis* Sonn.) with machine learning techniques**

Zuanxian Su^1^, Lunyang Liu^3^, Shanguo Ou^4,5^, Xiaodan Peng^4^, Guixiang Zhang^4^, Yunqi Li ^2*^, Houbin Chen^1[1]*^

^1^Guangdong Litchi Engineering Research Center, College of Horticulture, South China Agricultural University, Guangzhou 510640, China

^2^Department of Polymer Materials and Engineering, College of Materials and Metallurgy, Guizhou University, Guiyang 550025, China

^3^Changchun Institute of Applied Chemistry, Chinese Academy of Sciences, Changchun 130022, China

^4^Guangzhou Ecological and Agricultural Meteorological Center，Guangzhou 511430, China

^5^GBA Meteorological Intelligent Equipment Research Center/GBA Academy of Meteorological Research，Guangzhou，510555,China

**Fig. A.1.** Preliminary comparison of the predictive ability of classification models for Flr.State. RF: Random Forest algorithm, LDA: Linear Discriminant Analysis algorithm, SVM: Support Vector Machine algorithm, CART: Classification and Regression Trees algorithm, KNN: K-Nearest Neighbor algorithm.

| A   | B   |
| --- | --- |

**Fig. A.2.** The change of Accuracy and error of Random Forest classification algorithm. A, the change of accuracy when selecting different number of features. B, the change of error when selecting different number of trees.

**Fig. A.3.** The correlation matrix for the features against Flr.State and Flr.Rate, Spearman (CORR_sp_, upper triangle) and Pearson (CORR_Rp_, lower triangle) correlation coefficients are shown based on the 5-day data set. The positive/negative correlation and the coefficients are indicated by the color bar, green numbers and the size of circles.

1. *Abbreviations:* %IncMSE, Per cent increase in mean squared error; Achil.(5-35), The accumulation of chilling hours beneath the critical air temperatures indicated in parentheses in the process of flower induction; Achil.Max.(5-35), The accumulation of chilling per day about the maximum air temperature beneath the critical air temperatures indicated in parentheses in the process of flower induction; Achil.Mean.(5-35), The accumulation of chilling per day about the mean air temperature beneath the critical air temperatures indicated in parentheses in the process of flower induction; Achil.Min.(5-35), The accumulation of chilling per day about the minimum air temperature beneath the critical air temperatures indicated in parentheses in the process of flower induction; ANOVA, Analysis of variance; Athrm.(5-35), The accumulation of heat hours above the critical air temperatures in parentheses in the process of flower induction; Athrm.Max.(5-35), The accumulation of heat per day about the maximum air temperature above the critical temperatures in parentheses in the process of flower induction; Athrm.Mean.(5-35), The accumulation of heat per day about the mean air temperature above the critical temperatures in parentheses in the process of flower induction; Athrm.Min.(5-35), The accumulation of heat per day about the minimum air temperature above the critical temperatures in parentheses in the process of flower induction; AUC, The area under the receiver operating characteristic curve; CART, Classification and Regression Trees algorithm; CORR_p_, Pearson correlation coefficient; CORR_Sp_, Spearman correlation coefficient; Cul, Cultivar; Cul.FZX, Dummy variables about cultivar 'feizixiao' ; Cul.GW, Dummy variables about cultivar 'guiwei' ; Cul.HZ, Dummy variables about cultivar 'huaizhi'; Cul.NMC, Dummy variables about cultivar 'nuomici'; CV, Coefficient variation; d, Day; DFI, The duration of flower induction; DOFE, The date of floral white millet emergence; DOFM, The date of the last autumn flush maturation; DOY, Day of year; Flr.Good, Flowrering rate ≥75%; Flr.Poor, Flowrering rate <75%; Flr.Rate, Flower rate, Flr.State, Flowrer state; FN, False negative; FP, False positive; FZX, 'Feizixiao' ; GW, 'Guiwei' ; h, Hour; Humi.Mean, Mean atmospheric relative humidity; HZ, 'Huaizhi' ; KNN, K-NearestNeighbor algorithm; LDA, Linear discriminant analysis algorithm; MAE, Mean absolute error; Mean.Humi, The mean atmospheric relative humidity per hour in the process of flower induction; Mean.Humi.Max, The mean of max atmospheric relative humidity per day in the process of flower induction; Mean.Humi.Mean, The mean of mean atmospheric relative humidity per day in the process of flower induction; Mean.Humi.Min, The mean of minimum atmospheric relative humidity per day in the process of flower induction; Mean.Temp, The mean temperature per hour in the process of flower induction; Mean.Temp.Max, The mean of max temperature per day in the process of flower induction; Mean.Temp.Mean, The mean of mean temperature per day in the process of flower induction; Mean.Temp.Min, The mean of minimum temperature per day in the process of flower induction; NMC, 'Nuomici' ; NOF, Number of autumn flushes; RF, Random forest algorithm; RMSE, Root mean square error; ROC, Receiver operating characteristic curve; R_p_^2^, The coefficient of determination; SD, Standard deviation; SE , Standard error; SHAP, SHapley Additive exPlanations; Slneg.Temp, The sum of the rate of temperature drop per hour in the process of flower induction; Slneg.Temp.Max, The sum of the rate of maximum temperature drop per day in the process of flower induction; Slneg.Temp.Mean, The sum of the rate of mean temperature drop per day in the process of flower induction; Slneg.Temp.Min, The sum of the rate of minimum temperature drop per day in the process of flower induction; Slpos.Temp, The sum of the rate of temperature rise per hour in the process of flower induction; Slpos.Temp.Max, The sum of the rate of max temperature rise per day in the process of flower induction; Slpos.Temp.Mean, The sum of the rate of mean temperature rise per day in the process of flower induction; Slpos.Temp.Min, The sum of the rate of min temperature rise per day in the process of flower induction; SVM, Support Vector Machine algorithm,; Temp.Mean, Mean temperature; TN, True negative; TP, True positive; Year.Even, Even year; Year.Odd, Odd year.

   ^*^Corresponding author. Guangdong Litchi Engineering Research Center, College of Horticulture, South China Agricultural University, Guangzhou 510642, China (H. Chen); Department of Polymer Materials and Engineering, College of Materials and Metallurgy, Guizhou University, Guiyang 550025, China (Y. Li).

   *E-mail address*: [hbchen@scau.edu.cn](mailto:hbchen@scau.edu.cn) (Houbin Chen); liyq[@gzu.edu.cn](mailto:@gzu.edu.cn) (Yunqi Li).
